# Fetal Growth Acceleration—Current Approach to the Big Baby Issue

**DOI:** 10.3390/medicina57030228

**Published:** 2021-03-02

**Authors:** Jan Modzelewski, Anna Kajdy, Katarzyna Muzyka-Placzyńska, Dorota Sys, Michał Rabijewski

**Affiliations:** Department of Reproductive Health, Centre of Postgraduate Medical Education, 01-004 Warsaw, Poland; jmodzelewski@cmkp.edu.pl (J.M.); katarzyna.myzyka-placzynska@cmkp.edu.pl (K.M.-P.); dsys@cmkp.edu.pl (D.S.)

**Keywords:** diabetes, gestational, diagnostic techniques, obstetrical and gynecological, fetal growth acceleration, fetal macrosomia, large-for-gestational-age, obstetrics, pregnancy complications, stillbirth

## Abstract

*Background and Objectives*: Fetal overgrowth is related to many perinatal complications, including stillbirth, cesarean section, maternal and neonatal injuries, and shoulder dystocia. It is related to maternal diabetes, obesity, and gestational weight gain but also happens in low-risk pregnancies. There is ongoing discussion regarding definitions, methods of detection, and classification. The method used for detection is crucial as it draws a line between those at risk and low-risk popula-tions. *Materials and Methods*: For this narrative review, relevant evidence was identified through PubMed search with one of the general terms (macrosomia, large-for-gestational-age) combined with the outcome of interest. *Results*: This review summarizes evidence on the relation of fetal overgrowth with stillbirth, cesarean sections, shoulder dystocia, anal sphincter injury, and hem-orrhage. Customized growth charts help to detect mothers and fetuses at risk of those complica-tions. Relations between fetal overgrowth and diabetes, maternal weight, and gestational weight gain were investigated. *Conclusions*: a substantial proportion of complications are an effect of the fetus growing above its potential and should be recognized as a new dangerous condition of Fetal Growth Acceleration.

## 1. Introduction

Complications of pregnancy have a long-term effect on both the mother and baby; therefore, maternal and neonatal health is an essential public health issue. Fetal growth is a clinical proxy for fetal wellbeing. While one should expect that both ends of the growth spectrum have some kind of underlying pathology, only small for gestational age and fetal growth restriction have established definitions [1]. The other end of the spectrum, being too large, does not have a unified definition. Macrosomia is usually defined as an overgrowth of a fetus beyond a fixed cut-off value, while large-for-gestational age (LGA) is generally defined as being larger than the 90th centile. The most commonly cited macrosomia values are between 4000 and 4500 g [2].

On the other hand, neither of those definitions reflects the aspect of growth velocity. If the smaller growth spectrum is defined as fetal growth restriction (FGR), could we talk about fetal growth acceleration (FGA)? The latest definition of FGR includes the crossing of centiles in the late third trimester. Could this be the case in overgrowth as well? Secondly, could this be a potential risk factor for an abnormal perinatal outcome?

This review aims to summarize evidence on the consequences of fetal overgrowth, its relation with diabetes, and to propose a novel approach to the big baby issue.

## 2. Material and Methods

This is a narrative review. Articles relevant to this review were identified through a PubMed search with one of the general terms (macrosomia, large-for-gestational-age) combined with the outcome of interest. Systematic reviews, meta-analyses, and large-scale registers or cohort studies were included. Languages were restricted to English or Polish; no time restriction was made, but due to possible practice changes, newer publications on the same topic were preferred over older ones. 

For all results retrieved by the search, the title or title/abstract was scanned. In total, 65 full texts were read, and 41 relevant papers included. The fact that the study supported or did not support a hypothesis of this review did not affect the decision to include the study.

## 3. Results

### 3.1. Diabetes, Prepregnancy Weight, Gestational Weight Gain, and Genetic Disorders—Risk Factors for Fetal Overgrowth

There are several risk factors of extensive fetal growth, including diabetes, pre-pregnancy and gestational, maternal prepregnancy weight, and gestational weight gain.

Gestational diabetes mellitus (GDM) is defined as diabetes first diagnosed during pregnancy. Pregnancy physiologically results in higher insulin resistance. In women with GDM, this process is pronounced by pathological changes in both the mother and placenta. One of them is the disfunction of β-cells and higher maternal insulin resistance in pregnancy. The mother’s glucose transporter 4 (GLUT4) signaling becomes altered without changing receptor density. As a result, maternal glucose uptake is reduced to 54% compared to a normal pregnancy. In contrast, placental glucose uptake is increased [3]. As a result, the fetus produces more insulin and insulin-related growth factor-1, potent anabolic substances, leading to fetal overgrowth [4].

Pre-pregnancy diabetes, both type 1 and type 2, have an even more significant impact on fetal growth than gestational diabetes. Maternal hyperglycemia causes excessive glucose transport to the placenta, fetal hyperinsulinemia, and excess insulin growth factor-1 secretion. Glucose variability has a more substantial effect on fetal growth than basal glycemic levels reflected by HbA1_c_ levels. On the other hand, studies on optimal glycemic control showed a reduction in fetal overgrowth, but fetal growth was still altered. Further studies showed a relation between amino acid levels and advanced maternal age, leptin, fatty acids, especially triglycerides, and fetal overgrowth [5]. Surprisingly, despite more inadequate metabolic control reflected by HbA1_c_ levels, women with type 1 diabetes have better outcomes in perinatal mortality and congenital malformations than women with type 2 diabetes [6].

Gestational weight gain and pre-pregnancy BMI both impact fetal growth. A large metanalysis of individual patient data showed that the risk of having an LGA neonate grows substantially in women with higher pregestational BMI and in those who had excessive weight gain in pregnancy [7]. Both factors affect fetal growth independently and have an additive effect. Similar results were observed in other recent studies [8,9]. 

Macrosomia has consequences for both the mother and child. Most of all, excess fetal weight leads to an increased risk of hypoxia, postpartum hemorrhage (PPH), cesarean section, or instrumental delivery. Shoulder dystocia occurs more often, leading to fractures, brachial plexus injury, and obstetric anal sphincter injury (OASIS) [10,11]. 

Different factors involved in fetal overgrowth trigger specific growth patterns in the fetus. Children of diabetic mothers have more adipose tissue, lower fat-free body mass, and altered anthropometric measurements, especially higher fat deposition in the body’s upper half [12]. Higher bisacromial diameter was found in children of diabetic mothers and has been confirmed as a risk factor of shoulder dystocia [13].

Genetic disorders may also present with fetal overgrowth as in Beckwith–Wiedemann, Simpson–Golabi–Behmel, Weaver, familial hyperinsulinism, Elejalde, Costello, Cantu, and Perlman syndromes. Fetal overgrowth could also be present in Pallister-Kilian, Sotos, and Malan syndromes, but extensive fetal growth is less common [14,15]. Pallister–Kilian syndrome is often mentioned as an overgrowth syndrome, but only up to 40% of patients have generalized or segmental overgrowth [15].

### 3.2. Detection of Fetal Overgrowth

It is important to know the differences between the charts used to assess fetal growth. Descriptive reference curves describe growth in the given population and time. They are usually drawn in a retrospective manner and rarely have high methodological quality. Prescriptive standard curves show fetuses’ growth under optimal conditions, excluding any that could impact fetal growth. Prescriptive standard curves are usually drawn prospectively and have higher methodological quality. Some prescriptive standards are established internationally for large and diverse populations.

On the contrary, customized standards are an attempt to describe fetal growth on the individual level, taking ethnicity, parity, maternal weight and height, and fetal sex into account [16,17]. There is an ongoing discussion in the literature on which growth charts should be used. Choosing one chart over another changes LGA rates, reclassifies some of the fetuses, and therefore changes the association of LGA with pregnancy complications [18]. 

Our group has proven that SGA detection is the key to success, and it is probable that the same applies to LGA [19]. Different strategies were applied to optimize LGA detection. A comparison of serial scans and growth velocity with single measurements done by several groups shows that single measurement performed better in terms of sensitivity, especially if made close to delivery [20,21]. On the other hand, analysis of growth velocity between first- and second-trimester ultrasound scans could help predict the most severe cases of LGA > 97th centile and macrosomia. According to a paper by Simic et al., if growth acceleration equals or exceeded seven days of ultrasound estimated gestational age between first (11–14 weeks) and early second (18–20 weeks) scans, aOR for LGA and macrosomia was 2.27 (95% CI 1.49–3.45) [22]. Other strategies evaluated for macrosomia prediction were anthropometric measurements in the first trimester, maternal characteristic, fetal measurements (crown-rump length, nuchal translucency—NT, fetal heart ratio, first-trimester umbilical vein blood flow) and biochemical markers (placenta growth factor—PLGF, irisin, Fetuin-A, pregnancy-associated plasma protein A-PAPP-A, and free beta-hCG), which have proven some usefulness but require further studies [23,24,25,26,27]. The highest reported AUC was 0.818 for the biochemical marker irisin, 0.79 for the model consisting of BMI, parity, PAPP-A MoM, umbilical vein blood flow, and 0.727 for maternal factors, nuchal translucency, maternal serum free beta-hCG, and PAPP-A. In those studies, the reported AUC of prediction only by maternal and pregnancy characteristics was 0.657–0.715.

### 3.3. Complications

#### 3.3.1. Stillbirth

Whether accelerated fetal growth is associated with stillbirth is a matter of controversy. In a study by Wood and Tang based on a Canadian birth registry of 696,461 births, including 3275 stillbirths, no relationship was found between LGA and stillbirth [28]. The authors used the Canadian population, US population, and ultrasound growth norms, finding no relationship between norms used and outcome. 

Mecacci et al. conducted a case-control trial of stillbirth in two Italian tertiary care centers. In total, 175 stillbirth cases and 586 controls were analyzed using customized norms. Results showed a U-shaped stillbirth risk curve with the highest stillbirth risk in the lowest and highest weight centiles. LGA > 90th centile was associated with a 1.99 increase in stillbirth risk [29]. 

A case-control study conducted by Bukowski et al. evaluated 527 stillbirths and 1821 matched controls from five US geographic areas using population, ultrasound, and customized standards. They found that LGA was strongly associated with stillbirth when evaluated using ultrasound (aOR 3.71) and customized norms (aOR 2.57), but not population norms [30].

Another comparison of different growth charts and risk of perinatal complications, including perinatal mortality, was made by Sjaarda et al., showing that LGA increases the risk of stillbirth regardless of the growth chart used, but custom methods had higher PPV and but lower sensitivity; in the clinical setting, using a customized standard would miss more cases of LGA stillbirths, but those identified would be those in fact in danger [31].

A comparison between global Intergrowth-21st [32] and the customized growth chart done by Francis et al. showed an increased risk of stillbirth in a small-for-gestational-age group when using a customized growth chart. Results for LGA showed that using Intergrowth-21st growth chart LGA is a protective factor for stillbirth and a risk factor when using customized growth charts, but the results were not statistically significant [33].

A study comparing the usage of the population and partially customized (using parity, fetal sex, maternal weight, and height) growth charts on data collected from Scottish medical databases by Iliodromiti et al., included 979,912 term pregnancies from 1992–2010 and showed that LGA neonates by customized growth charts had a higher risk of stillbirth (aOR 1.29; LGA birthweight > 90th centile; aOR 1.25 LGA birthweight > 85th centile). This effect was not visible when using population growth charts [34].

Risk factors of stillbirth related to macrosomia were studied by Tam Giao Cung et al. The researchers analyzed all births from a major hospital in Nablus, Palestine. The cohort consisted of 5644 births and 5782 babies and included 41 stillbirths. Macrosomia was defined as weight 4500 g and higher. Adjusted OR for stillbirth in macrosomia was aOR 6.3 compared to a reference birthweight of 2500–4499 g [35]. 

In a recent study by Salihu et al., the authors analyzed national registers of 111,166,370 terms from 1987–2017. Macrosomia was defined as weight > 4000 g and was further divided into subgroups of grade one—4000–4499 g, grade 2—4500–4999 g, and grade 3—>5000 g. The authors observed a substantial fall in stillbirth rates during the study period in both macrosomic and normal weight fetuses. Grade 1 macrosomia was not a stillbirth risk factor (0.95 stillbirths per 1000 pregnancies), while grade 2 macrosomia increased the risk 2-fold (2.43/1000) and grade 3 had a stillbirth rate (13.03/1000)—similar to that of low birthweight fetuses (15.54/1000) [36]. 

Some authors suggest that the macrosomia cut-off should be race-specific, which brings some analogy to growth chart customization. Research of linked US datasets of birth and infant death (30,831,694 live births and 38,053 stillbirths) by Ye et al. showed that the optimal cut-off for Caucasians is 4500 g, but that for Blacks and Hispanics should be lower, by 200 g. Cut-off points of 4500 g and 4300 g were chosen because the indicated OR for perinatal mortality reached a pre-defined value of 2.0. Other races were not analyzed as they were not represented in sufficient numbers. The authors made adjustments for pregnancy complications, including diabetes, in their analysis [37].

Similar results indicating that macrosomia or LGA is a risk factor of stillbirth were reported by Agbozo et al. (aOR 2.4; macrosomia > 4000 g) [38], Contag et al. [39] (HR 2.2; LGA EFW > 95th centile)*,* Lavin et al. [40] (RR 3.4–6.6; EFW > 90th centile), and Moraitis et al. [41] (OR 2.2; EFW > 98th centile). All LGA study groups were defined using population growth charts.

#### 3.3.2. Shoulder Dystocia, OASIS, PPH

Shoulder dystocia may occur even if a neonate is small-for-gestational-age, but the risk grows with increasing birth weight [42]. As LGA categories by any standard and macrosomia overlap within higher birthweights, it is difficult to choose the best tool for predicting these complications. 

A direct comparison of population and customized growth charts for LGA and macrosomia by Larkin et al. showed a significant risk of shoulder dystocia, OASIS, and PPH. Still, none was superior to others [43]. The analysis was performed on 32271 cases from a single center, including 1256–2002 (depending on the method used) cases of fetal overgrowth. 

In a previously mentioned paper by Sjaada et al., the authors compared different growth charts. In an analysis of 168,945 births from 12 research centers, the authors used population growth charts, customized growth charts from Gardosi [44], and their model. By either method but not population growth charts, mothers of LGA neonates identified by customized growth charts had a greater risk of OASIS. Additionally, LGA determined by the authors’ model had a greater risk of shoulder dystocia. The risk of PPH was similar in both groups. LGA identified by population growth chart only had a lower risk of OASIS and shoulder dystocia than LGA identified by any customized method.

The risk of PPH in mothers of macrosomic non-LGA babies (by customized growth chart) was analyzed by Pasupathy et al. in 2668 cases [45]. The authors showed that mothers of neonates with birth weight > 4000 g but non-LGA by customized growth chart had an insignificantly higher risk of postpartum hemorrhage. On the other hand LGA with birthweight < 4000 g was a risk factor of PPH (aOR 2.7 [95% CI 1.2–6.2]). Neither population nor customized growth charts resulted in a more significant RR for PPH in direct comparison. 

Vieira et al., had a different approach to choosing the right tool for assessing the risk of complications [46]. Based on the Swedish Medical Birth Registry, a population of 212,101 term births was chosen. Using a fixed 10% false-positive rate, the authors found growth chart-specific cut-off points for perinatal complications, including PPH, cesarean section, neonatal complications, and OASIS, while achieving similar sensitivity. On the other hand, customized charts performed better in terms of perinatal mortality of LGA babies.

The rate of complications between term non-macrosomic LGA and AGA was compared by Doty et al. based on US birth certificates and US Vital Statistic databases. Macrosomia was defined as birthweight > 4000 g; LGA was assessed by a population standard. Of 3,917,831 births, only 50,630 (1.3%) were non-macrosomic LGA. The authors concluded that non-macrosomic LGA had a greater risk of combined neonatal (aOR 1.47) and maternal (aOR 1.40) morbidity, including a higher risk of maternal transfusion, maternal unplanned operating room procedures, need of assisted ventilation of neonate, fetal significant birth injury, or Apgar score < 5 at 5 min [47].

A meta-analysis comparing population and customized growth charts by Chossi et al. did not report that LGA neonates and mothers had a statistically significant risk of intrauterine death, admission to neonatal intensive care unit, fetal postpartum hypoglycemia, or maternal OASIS, with both growth charts used. Both population and customized growth charts showed an increased risk of shoulder dystocia [48].

#### 3.3.3. Cesarean Section

The mode of delivery of LGA fetuses is of significant concern to healthcare practitioners. The right choice of the at-risk population will result in the lowest possible rate of complications.

Different methods of customization could have different effects on intrapartum complications and the need for emergency cesarean section. Pritchard et al., compared the cesarean section’s risk between AGA babies and those classified as LGA by population, height-only customized, and height and weight customized growth charts. In total, 38,246 births were analyzed, and 1917 babies were LGA by population charts, 1754 LGA by height customization, and 1904 LGA by weight and height customization. Accordingly, 290, 263, and 413 were considered LGA by only one growth chart. Customization by height only showed the highest correlation with emergency cesarean section (OR 4.64; 95% CI 3.22–6.76) while population chart LGA did not show such a correlation (OR 1.46; 95% CI 0.70–1.88), with a small difference in mean birth weight (4140 us. 4015 g). LGA height, the only customization, showed a better correlation with the risk of cesarean section compared to weight-height customization charts (OR 1.85, 95% CI 1.32–2.61). When fetuses identified by only one growth chart were taken into consideration, women with LGA neonates by height-only customization were at the greatest risk for emergency cesarean section (OR 1.85, 95% CI 1.32–2.61). The overall rate of cesarean sections, both emergency and planned, was highest in the LGA customized by height only group (61.4%). In comparison, LGA by population charts had a value of only 34.5%; LGA by weight–height customization had a cesarean section rate of 41.6%; and AGA, 27.3% [49]. 

Customized LGA by both methods showed greater OR for cesarean section than population growth charts in the study by Sjaarda et al. [31], but Larkin et al. [43] did not show any differences in cesarean section in LGA and macrosomia, regardless of the method used.

## 4. Discussion

Not surprisingly, complications of fetal overgrowth are rising with increasing fetal weight. However, complications occur even if the weight of the fetus is below the defined centile or fixed cut-off point. Evidence regarding stillbirth is confusing, but the majority of papers show that there is a link between fetal overgrowth and macrosomia. Recognition of fetal overgrowth as a risk factor of shoulder dystocia, OASIS, and PPH is well established. When it comes to cesarean section, maternal height-customized fetal overgrowth diagnosis was the greatest risk factor. All evidence comes from registry analysis or cohort studies; there were no randomized studies to evaluate the methods of fetal overgrowth diagnosis directly. Even without any RCT, the available evidence described above should be considered in everyday practice and by policymakers.

Limitations of this study should be acknowledged. Firstly, the risk of bias was not assessed, including publication and reporting bias. None of the presented studies was randomized, and most were registry-based, with limitations specific to this kind of study. All identified research was included, but only one database was searched. Methodological differences between studies often make direct comparison impossible.

**Figure 1:**
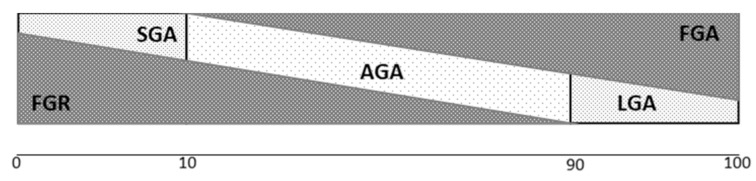
Relationship between AGA—Appropriate for Gestational Age, LGA—large for gestational age, SGA—Small for Gestational Age, and FGA—Fetal Growth Acceleration.

In our opinion, this phenomenon of fetal overgrowth needs to be recognized in a similar way that pathological smallness (FGR) and constitutional smallness (small for gestational age) are recognized [50]. Therefore, we propose defining Fetal Growth Acceleration (FGA) as a new and pathological condition distinct from LGA and macrosomia. FGA fetuses’ growth exceeds their genetic optimal growth and, what is most important, exceeds placental capacity. Not all FGR have to be necessary SGA, in a similar way as not all FGA have to be macrosomic or LGA, as shown in Figure 1. Therefore, we want to propose the definition of FGA, knowing that it needs to be proven before adopting it into widespread clinical use. The proposal of the definition is presented in Table 1. 

Usage of customized charts could help detect those FGA cases that are missed by regular charts, but not all the cases; therefore, new tools need to be developed.

## 5. Conclusions

Contemporary research shows that the method of fetal overgrowth assessment is of great importance, with emphasis on customization. Customization helps to optimize the choice of at-risk fetuses and therefore facilitates the delivery of an appropriate intervention. There is probably a need for more than one method of customization depending on which complications aim to be avoided. Hopefully, fetal overgrowth is receiving more attention from the scientific community, promising more progress in this field soon.

## Figures and Tables

**Table 1:** Proposal of FGA definition.

Sole Criterium	Multiple Criteria
LGA > 97th centile on customized growth charts	LGA > 90th centile OR crossing centiles by >2 quartiles on customized growth charts AND any of below: Umbilical artery pulsatility index PI > 95th centile Middle cerebral artery PI < 5th centile CPR < 5th centile

FGA—fetal growth acceleration; LGA—large for gestational age; Cerebro-placental ratio—CPR.

## Abbreviations

AGA—Appropriate for Gestational Age; Body Mass Index–BMI; Cerebro-placental ratio–CPR; FGA—Fetal Growth Acceleration; FGR–Fetal Growth Restriction; Gestational diabetes mellitus–GDM; Glucose Transporter 4-GLUT4; LGA—large for gestational age; nuchal translucency–NT; Obstetric Anal Sphincter Injury–OASIS; Placenta Growth Factor–PLGF; Postpartum Hemorrhage–PPH; Pregnancy-associated Plasma Protein A-PAPP-A; Pulsatility index–PI; SGA—Small for Gestational Age.
